# 3-[(3,5-Dichloro­anilino)carbon­yl]propionic acid

**DOI:** 10.1107/S1600536809010319

**Published:** 2009-03-25

**Authors:** B. Thimme Gowda, Sabine Foro, B. S. Saraswathi, Hiromitsu Terao, Hartmut Fuess

**Affiliations:** a Department of Chemistry, Mangalore University, Mangalagangotri 574 199, Mangalore, India; b Institute of Materials Science, Darmstadt University of Technology, Petersenstrasse 23, D-64287 Darmstadt, Germany; c Faculty of Integrated Arts and Sciences, Tokushima University, Minamijosanjima-cho, Tokushima 770-8502, Japan

## Abstract

In the crystal structure of the title compound, C_10_H_9_Cl_2_NO_3_, the conformations of the amide O atom and the carbonyl O atom of the acid segment are *anti* to the H atoms of the adjacent –CH_2_ groups. The C=O and O—H bonds of the acid group are in relatively rare *anti* positions with respect to each other. This is an obvious consequence of the concerted effects of both the all-*anti* mol­ecular conformation and the intermolecular hydrogen bond donated to the amide carbonyl group. In the crystal, mol­ecules are packed into infinite chains through inter­molecular N—H⋯O and O—H⋯O hydrogen bonds.

## Related literature

For the effect of ring and side-chain substitutions on the structures of amide compounds, see: Gowda *et al.* (2009 ▶). For the packing of mol­ecules involving dimeric hydrogen-bonded association of each carboxyl group with a centrosymmetrically related neighbor, see: Jagannathan *et al.* (1994 ▶). For the various modes of inter­linking carboxylic acids by hydrogen bonds, see: Leiserowitz (1976 ▶).
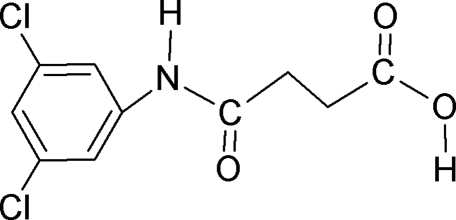


## Experimental

### 

#### Crystal data

C_10_H_9_Cl_2_NO_3_
*M* *_r_* = 262.08
Monoclinic, 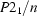

*a* = 7.350 (1) Å
*b* = 10.318 (2) Å
*c* = 15.031 (3) Å
β = 99.44 (2)°
*V* = 1124.5 (3) Å^3^
*Z* = 4
Cu *K*α radiation
μ = 5.15 mm^−1^
*T* = 299 K
0.48 × 0.30 × 0.28 mm

#### Data collection

Enraf–Nonius CAD-4 diffractometer
Absorption correction: ψ scan (North *et al.*, 1968 ▶) *T*_min_ = 0.142, *T*_max_ = 0.242
4204 measured reflections
2005 independent reflections
1794 reflections with *I* > 2σ(*I*)
*R*_int_ = 0.111
3 standard reflections frequency: 120 min intensity decay: 1.0%

#### Refinement

*R*[*F* ^2^ > 2σ(*F* ^2^)] = 0.058
*wR*(*F* ^2^) = 0.170
*S* = 1.13
2005 reflections
151 parameters
1 restraint
H atoms treated by a mixture of independent and constrained refinement
Δρ_max_ = 0.37 e Å^−3^
Δρ_min_ = −0.54 e Å^−3^

### 

Data collection: *CAD-4-PC* (Enraf–Nonius, 1996 ▶); cell refinement: *CAD-4-PC*; data reduction: *REDU4* (Stoe & Cie, 1987 ▶); program(s) used to solve structure: *SHELXS97* (Sheldrick, 2008 ▶); program(s) used to refine structure: *SHELXL97* (Sheldrick, 2008 ▶); molecular graphics: *PLATON* (Spek, 2009 ▶); software used to prepare material for publication: *SHELXL97*.

## Supplementary Material

Crystal structure: contains datablocks I, global. DOI: 10.1107/S1600536809010319/cs2111sup1.cif

Structure factors: contains datablocks I. DOI: 10.1107/S1600536809010319/cs2111Isup2.hkl

Additional supplementary materials:  crystallographic information; 3D view; checkCIF report

## Figures and Tables

**Table 1:** Hydrogen-bond geometry (Å, °)

*D*—H⋯*A*	*D*—H	H⋯*A*	*D*⋯*A*	*D*—H⋯*A*
N1—H1*N*⋯O3^i^	0.95 (4)	1.93 (4)	2.857 (3)	167 (3)
O2—H2*O*⋯O1^ii^	0.82 (2)	1.85 (2)	2.656 (3)	170 (4)

Symmetry codes: (i) 
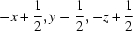
; (ii) 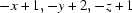.

**Fig. 1.**
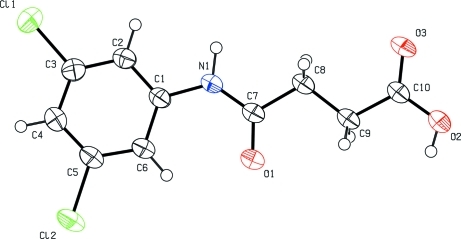
Molecular structure of the title molecule with atom labeling. Displacement ellipsoids are at the 50% probability level, H atoms represented as small spheres of arbitrary radii.

**Fig. 2.**
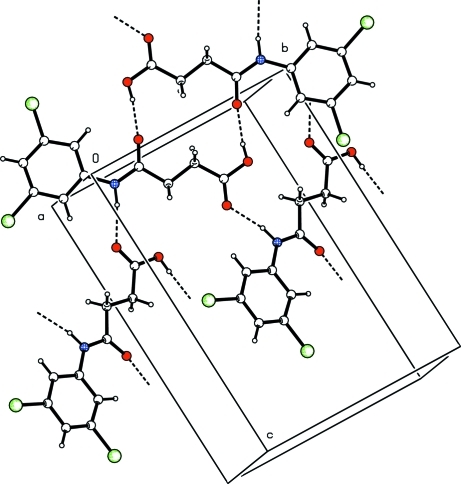
Molecular packing detail in the title crystal with hydrogen bonds shown as dashed lines.

## supplementary crystallographic information

### Comment

The amide moiety is an important constituent of many biologically significant compounds. As a part of studying the effect of ring and side chain substitutions on the structures of this class of compounds (Gowda *et al.*, 2009), we have determined the crystal structure of *N*-(3,5-dichlorophenyl)-succinamic acid (N35DCPSA, systematic name: 3-[(3,5-dichloro)-aminocarbonyl]propionic acid). The conformations of N—H and C=O bonds in the amide segment of the structure are *anti* to each other and those of the amide O atom and the carbonyl O atom of the acid segment are also *anti* to the H atoms attached to the adjacent C atoms (Fig.1). Further, C=O and O—H bonds of the acid group are *anti* to each other, contrary to the more general *syn* conformation observed for C=O and O—H bonds of the acid group e.g. *N*-(2,6-dimethylphenyl)- succinamic acid (N26DMPSA, Gowda *et al.*, 2009). The various modes of interlinking carboxylic acids by hydrogen bonds is described elsewhere (Leiserowitz, 1976). The packing of molecules involving dimeric hydrogen bonded association of each carboxyl group with a centrosymmetrically related neighbor has also been observed (Jagannathan *et al.*, 1994). In the present study, the rare *anti* conformation of the C=O and O—H bonds of the acid group has been observed. The torsional angles of the groups, C2—C1—N1—C7, C6—C1—N1—C7, C1—N1—C7—C8, C1—N1—C7—O1, N1—C7—C8—C9, C7—C8—C9—C10, O1—C7—C8—C9, C8—C9—C10—O2 and C8—C9—C10—O3 in the side chain of N35DCPSA are -180.0 (3)°, -1.3 (4)°, -174.4 (3)°, 4.3 (5)°, 178.9 (2)°, -175.5 (2)°, 0.4 (2)°, 175.0 (3)° and -5.3 (5)°, respectively, compared to the corresponding values of 114.1 (2)°, -66.5 (2)°, -176.2 (1)°, 2.0 (3)°, -145.4 (2)°, -175.5 (1)°, 36.3 (2)°, -161.1 (2)° and 19.1 (3)°, respectively, for N26DMPSA. The N—H···O and O—H···O intermolecular hydrogen bonds pack the molecules into infinite chains in the structure (Table 1, Fig.2).

### Experimental

The solution of succinic anhydride (0.025 mol) in toluene (25 ml) was treated dropwise with the solution of 3,5-dichloroaniline (0.025 mol) also in toluene (20 ml) with constant stirring. The resulting mixture was stirred for about one hour and set aside for an additional hour at room temperature for the completion of reaction. The mixture was then treated with dilute hydrochloric acid to remove the unreacted 3,5-dichloroaniline. The resultant solid *N*-(3,5-dichlorophenyl)-succinamic acid was filtered under suction and washed thoroughly with water to remove the unreacted succinic anhydride and succinic acid. It was recrystallized to constant melting point from ethanol. The purity of the compound was checked by elemental analysis and characterized by its infrared and NMR spectra. The single crystals used in X-ray diffraction studies were grown in an ethanol solution by slow evaporation at room temperature.

### Refinement

The N-bound and O-bound H atoms were located in a difference map. The position of the N-bound H atom was refined with N—H = 0.95 (4) Å and that of the O—H was refined with a distance restrained to 0.82 (2) Å. The other H atoms were positioned with idealized geometry using a riding model [C—H = 0.93–0.97Å]. All H atoms were treated with isotropic displacement parameters (set to 1.2 times of the *U*_eq_ of the parent atom).

### Crystal data

**Table:**

C_10_H_9_Cl_2_NO_3_	*F*(000) = 536
*M**_r_* = 262.08	*D*_x_ = 1.548 Mg m^−^^3^
Monoclinic, *P*2_1_/*n*	Cu *K*α radiation, λ = 1.54180 Å
Hall symbol: -P 2yn	Cell parameters from 25 reflections
*a* = 7.350 (1) Å	θ = 7.4–20.6°
*b* = 10.318 (2) Å	µ = 5.15 mm^−^^1^
*c* = 15.031 (3) Å	*T* = 299 K
β = 99.44 (2)°	Prism, colourless
*V* = 1124.5 (3) Å^3^	0.48 × 0.30 × 0.28 mm
*Z* = 4	

### Data collection

**Table:**

Enraf–Nonius CAD-4 diffractometer	1794 reflections with *I* > 2σ(*I*)
Radiation source: fine-focus sealed tube	*R*_int_ = 0.111
graphite	θ_max_ = 67.1°, θ_min_ = 5.2°
ω/2θ scans	*h* = 0→8
Absorption correction: ψ scan (North *et al.*, 1968)	*k* = −12→12
*T*_min_ = 0.142, *T*_max_ = 0.242	*l* = −17→17
4204 measured reflections	3 standard reflections every 120 min
2005 independent reflections	intensity decay: 1.0%

### Refinement

**Table:**

Refinement on *F*^2^	Primary atom site location: structure-invariant direct methods
Least-squares matrix: full	Secondary atom site location: difference Fourier map
*R*[*F*^2^ > 2σ(*F*^2^)] = 0.058	Hydrogen site location: inferred from neighbouring sites
*wR*(*F*^2^) = 0.170	H atoms treated by a mixture of independent and constrained refinement
*S* = 1.13	*w* = 1/[σ^2^(*F*_o_^2^) + (0.0804*P*)^2^ + 0.4071*P*] where *P* = (*F*_o_^2^ + 2*F*_c_^2^)/3
2005 reflections	(Δ/σ)_max_ = 0.009
151 parameters	Δρ_max_ = 0.37 e Å^−^^3^
1 restraint	Δρ_min_ = −0.54 e Å^−^^3^

### Fractional atomic coordinates and isotropic or equivalent isotropic displacement parameters (Å^2^)

**Table:**

	*x*	*y*	*z*	*U*_iso_*/*U*_eq_	
C1	0.2687 (3)	0.5234 (3)	0.51327 (16)	0.0360 (6)	
C2	0.1971 (3)	0.4036 (3)	0.48363 (18)	0.0390 (6)	
H2	0.1668	0.3863	0.4223	0.047*	
C3	0.1715 (4)	0.3108 (3)	0.54585 (19)	0.0415 (6)	
C4	0.2153 (4)	0.3313 (3)	0.63743 (19)	0.0453 (7)	
H4	0.1971	0.2676	0.6789	0.054*	
C5	0.2872 (4)	0.4502 (4)	0.66443 (18)	0.0453 (7)	
C6	0.3155 (4)	0.5480 (3)	0.60551 (17)	0.0435 (7)	
H6	0.3642	0.6276	0.6266	0.052*	
C7	0.3499 (3)	0.7371 (3)	0.45487 (16)	0.0374 (6)	
C8	0.3313 (4)	0.8125 (3)	0.36730 (17)	0.0421 (7)	
H8A	0.3996	0.7685	0.3263	0.051*	
H8B	0.2025	0.8148	0.3395	0.051*	
C9	0.4014 (4)	0.9480 (3)	0.38180 (17)	0.0447 (7)	
H9A	0.5322	0.9451	0.4055	0.054*	
H9B	0.3400	0.9891	0.4268	0.054*	
C10	0.3724 (4)	1.0293 (3)	0.29825 (18)	0.0436 (7)	
N1	0.2863 (3)	0.6158 (3)	0.44600 (15)	0.0403 (6)	
H1N	0.242 (4)	0.584 (4)	0.387 (3)	0.048*	
O1	0.4152 (3)	0.7867 (3)	0.52683 (13)	0.0543 (6)	
O2	0.4186 (4)	1.1531 (3)	0.30787 (14)	0.0622 (7)	
H2O	0.459 (5)	1.167 (5)	0.3609 (15)	0.075*	
O3	0.3114 (4)	0.9893 (3)	0.22424 (13)	0.0620 (7)	
Cl1	0.07961 (12)	0.16217 (8)	0.50728 (6)	0.0580 (3)	
Cl2	0.34909 (13)	0.47994 (11)	0.77952 (5)	0.0696 (4)	

### Atomic displacement parameters (Å^2^)

**Table:**

	*U*^11^	*U*^22^	*U*^33^	*U*^12^	*U*^13^	*U*^23^
C1	0.0448 (12)	0.0368 (17)	0.0252 (12)	0.0015 (11)	0.0026 (9)	0.0014 (11)
C2	0.0487 (13)	0.0390 (17)	0.0287 (12)	0.0004 (12)	0.0050 (10)	−0.0022 (11)
C3	0.0490 (13)	0.0379 (17)	0.0382 (14)	−0.0009 (12)	0.0089 (10)	−0.0034 (12)
C4	0.0589 (16)	0.044 (2)	0.0339 (14)	−0.0048 (13)	0.0098 (11)	0.0050 (12)
C5	0.0585 (15)	0.051 (2)	0.0265 (13)	−0.0048 (13)	0.0071 (10)	0.0019 (12)
C6	0.0590 (15)	0.0454 (19)	0.0254 (13)	−0.0089 (13)	0.0044 (10)	−0.0008 (12)
C7	0.0463 (12)	0.0401 (17)	0.0242 (11)	−0.0013 (12)	0.0007 (9)	0.0008 (11)
C8	0.0598 (15)	0.0408 (17)	0.0237 (12)	−0.0014 (13)	0.0007 (10)	0.0025 (11)
C9	0.0643 (15)	0.0440 (19)	0.0230 (12)	−0.0036 (14)	−0.0009 (10)	0.0038 (12)
C10	0.0611 (15)	0.0426 (18)	0.0252 (12)	−0.0021 (13)	0.0012 (10)	0.0043 (12)
N1	0.0602 (12)	0.0370 (15)	0.0220 (10)	−0.0033 (11)	0.0019 (8)	−0.0001 (9)
O1	0.0824 (13)	0.0483 (15)	0.0272 (10)	−0.0156 (11)	−0.0056 (9)	0.0019 (9)
O2	0.1047 (17)	0.0458 (15)	0.0289 (11)	−0.0123 (13)	−0.0107 (10)	0.0092 (10)
O3	0.1032 (17)	0.0545 (16)	0.0224 (10)	−0.0094 (13)	−0.0071 (10)	0.0031 (9)
Cl1	0.0808 (6)	0.0403 (6)	0.0533 (5)	−0.0134 (4)	0.0127 (4)	−0.0065 (3)
Cl2	0.1032 (7)	0.0795 (8)	0.0243 (4)	−0.0260 (5)	0.0056 (4)	0.0014 (3)

### Geometric parameters (Å, °)

**Table:**

C1—C2	1.388 (4)	C7—N1	1.335 (4)
C1—C6	1.396 (4)	C7—C8	1.516 (4)
C1—N1	1.411 (4)	C8—C9	1.494 (5)
C2—C3	1.372 (4)	C8—H8A	0.97
C2—H2	0.93	C8—H8B	0.97
C3—C4	1.378 (4)	C9—C10	1.496 (4)
C3—Cl1	1.737 (3)	C9—H9A	0.97
C4—C5	1.371 (5)	C9—H9B	0.97
C4—H4	0.93	C10—O3	1.202 (4)
C5—C6	1.381 (4)	C10—O2	1.323 (4)
C5—Cl2	1.742 (3)	N1—H1N	0.95 (4)
C6—H6	0.93	O2—H2O	0.82 (2)
C7—O1	1.221 (3)		
			
C2—C1—C6	120.0 (3)	N1—C7—C8	114.5 (2)
C2—C1—N1	116.5 (2)	C9—C8—C7	112.0 (2)
C6—C1—N1	123.5 (3)	C9—C8—H8A	109
C3—C2—C1	119.3 (2)	C7—C8—H8A	109
C3—C2—H2	120.3	C9—C8—H8B	109
C1—C2—H2	120.3	C7—C8—H8B	109
C2—C3—C4	122.6 (3)	H8A—C8—H8B	108
C2—C3—Cl1	118.5 (2)	C8—C9—C10	113.8 (2)
C4—C3—Cl1	118.9 (2)	C8—C9—H9A	109
C5—C4—C3	116.7 (3)	C10—C9—H9A	109
C5—C4—H4	121.7	C8—C9—H9B	109
C3—C4—H4	121.7	C10—C9—H9B	109
C4—C5—C6	123.7 (3)	H9A—C9—H9B	108
C4—C5—Cl2	118.5 (2)	O3—C10—O2	118.8 (3)
C6—C5—Cl2	117.8 (3)	O3—C10—C9	124.4 (3)
C5—C6—C1	117.7 (3)	O2—C10—C9	116.8 (3)
C5—C6—H6	121.1	C7—N1—C1	129.3 (2)
C1—C6—H6	121.1	C7—N1—H1N	118 (2)
O1—C7—N1	124.2 (3)	C1—N1—H1N	112 (2)
O1—C7—C8	121.3 (3)	C10—O2—H2O	109 (3)
			
O1—C7—C8—C9	0.2 (4)	O1—C7—N1—C1	4.3 (5)
N1—C7—C8—C9	178.9 (2)	C8—C7—N1—C1	−174.4 (3)
C7—C8—C9—C10	−175.5 (2)	C2—C1—N1—C7	180.0 (3)
C8—C9—C10—O3	−5.3 (5)	C6—C1—N1—C7	1.3 (4)
C8—C9—C10—O2	175.0 (3)		

### Hydrogen-bond geometry (Å, °)

**Table:**

*D*—H···*A*	*D*—H	H···*A*	*D*···*A*	*D*—H···*A*
N1—H1N···O3^i^	0.95 (4)	1.93 (4)	2.857 (3)	167 (3)
O2—H2O···O1^ii^	0.82 (2)	1.85 (2)	2.656 (3)	170 (4)

Symmetry codes: (i) −*x*+1/2, *y*−1/2, −*z*+1/2; (ii) −*x*+1, −*y*+2, −*z*+1.
